# Commentary: Sodium levels and immunotherapy efficacy in mRCC patients with bone metastases: sub analysis of the Meet-Uro 15 study

**DOI:** 10.3389/fimmu.2025.1555559

**Published:** 2025-02-12

**Authors:** Martina Catalano, Sara Elena Rebuzzi, Giuseppe Fornarini, Giandomenico Roviello

**Affiliations:** ^1^ Department of Health Sciences, Section of Clinical Pharmacology and Oncology, University of Firenze, Firenze, Italy; ^2^ Medical Oncology Unit, Ospedale San Paolo, Savona, Italy; ^3^ Medical Oncology Unit 1, Istituti di Ricovero e Cura a Carattere Scientifico (IRCCS) Ospedale Policlinico San Martino of Genova, Genova, Italy

**Keywords:** bone metastases, efficacy outcomes, sodium levels, renal cell carcinoma, immunotherapy

We are grateful to the correspondent for their thoughtful comments on our recently published study, “Sodium levels and immunotherapy efficacy in mRCC patients with bone metastases: sub analysis of the Meet-Uro 15 study.” We appreciate the opportunity to further discuss several key aspects of our work and the insightful suggestions for improving the robustness of our findings. Below, we address the concerns raised and offer clarification on the points mentioned.

We agree that metastatic renal cell carcinoma (mRCC) with bone metastases represents a unique subset of patients with particularly poor prognosis, and we acknowledge the importance of identifying prognostic factors to optimize treatment strategies. For this the focus of our study was on the prognostic role of natremia specifically in this patient population. In fact, in the MeetURO 15 study, we evaluated the prognostic role of natremia, including normonatremic patients with a cut-off of 140 mEq/L, in all patients with mRCC treated with nivolumab (1). The findings confirmed the negative prognostic role of sodium levels below 140 mEq/L. In this subanalysis, we aimed to determine whether sodium levels below 140 mEq/L retained their negative prognostic value specifically in RCC patients with bone metastases, obtaining confirmation (2).

Regarding the proposal to integrate inflammatory markers and to develop a multivariable prognostic scoring system similar to the Lung Immune Prognostic Index (LIPI) used in non-small cell lung cancer (NSCLC), we acknowledge the growing importance of incorporating systemic inflammatory markers such as the neutrophil-to-lymphocyte ratio (NLR) and lactate dehydrogenase (LDH) into prognostic models (3). Incorporating these variables may help to improve risk stratification and guide personalized therapeutic decisions for RCC patients, particularly those undergoing immunotherapy.

We also concur with the assertion that renal function, particularly serum creatinine levels, could play a role in influencing sodium levels and prognosis. The decision not to include creatinine in our initial analysis was based on the desire to isolate sodium as an independent variable, but we recognize that this exclusion may have introduced confounding factors. We agree that future analyses should adjust for renal function to better understand the relationship between hyponatremia and survival outcomes, especially considering the high prevalence of impaired renal function in RCC patients due to nephrectomy or nephrotoxic medications. This adjustment could lead to more precise conclusions about the prognostic significance of natremia in the context of renal impairment.

Finally, regarding the IMDC score, while it remains a widely utilized prognostic tool in mRCC, we agree with the suggestion that its applicability in the context of immunotherapy warrants further investigation. As immune checkpoint inhibitors become an integral part of mRCC treatment, the limitations of the IMDC score in capturing the dynamic nature of the tumor immune microenvironment are increasingly apparent. Developing new scoring systems that account for variables such as sodium and creatinine levels, as well as markers of immune activity and tumor-infiltrating lymphocytes, could significantly enhance the precision of prognostication in the immunotherapy era.

We appreciate the recognition of the novel contributions made by our study, particularly the targeted focus on RCC patients with bone metastases. Moving forward, we plan to pursue additional research that incorporates the suggestions provided, including broader cohort analyses and the integration of renal function parameters, to refine our understanding of the role of sodium in RCC prognosis.

## References

[1] Catalano M Rebuzzi SE Maruzzo M De Giorgi U Buti S Galli L. Sodium levels and outcomes in patients with metastatic renal cell carcinoma receiving nivolumab. JAMA Netw Open. (2023) 6(11):e2345185. doi: 10.1001/jamanetworkopen.2023.45185 38010650 PMC10682835

[2] Catalano M Rebuzzi SE Maruzzo M De Giorgi U Buti S Galli L. Sodium levels and immunotherapy efficacy in MRCC patients with bone metastases: sub analysis of meet-uro 15 study. Front Immunol. (2024) 15:1361010. doi: 10.3389/FIMMU.2024.1361010 39034992 PMC11257879

[3] Mezquita L Auclin E Ferrara R Charrier M Remon J Planchard D. Association of the lung immune prognostic index with immune checkpoint inhibitor outcomes in patients with advanced non-small cell lung cancer. JAMA Oncol. (2018) 4:351–7. doi: 10.1001/JAMAONCOL.2017.4771 PMC5885829 29327044

